# *ANOS1* variant and *PROK2* upregulation in familial Kallmann syndrome: integrative genetic, transcriptomic, and phenotypic study

**DOI:** 10.3389/fnmol.2026.1960672

**Published:** 2026-10-22

**Authors:** Katarzyna Bilińska, Angelika Gajewska, Karolina Hołub, Paweł Wojciechowski, Fernando de Castro, Magdalena Pasińska

**Affiliations:** 1 Department of Clinical Genetics, Faculty of Medicine, Collegium Medicum in Bydgoszcz, Nicolaus Copernicus University, Bydgoszcz, Poland; 2 Centro de Neurociencias Cajal-CSIC, Consejo Superior de Investigaciones Científicas-CSIC, Alcalá de Henares, Spain; 3 Institute of Computing Science, Poznan University of Technology, Poznan, Poland; 4 Institute of Bioorganic Chemistry, Polish Academy of Sciences, Poznan, Poland

**Keywords:** anosmia, anosmin-1, hypogonadotropic hypogonadism, Kallmann syndrome, neuron migration, olfactory dysfunction, prokineticin-2, RNA sequencing

## Abstract

Kallmann syndrome (KS) arises from impaired development of the olfactory–GnRH neuronal system, yet the molecular consequences of individual gene defects remain poorly defined. In particular, the link between primary genetic variants and downstream transcriptional changes affecting neurodevelopmental pathways remains poorly understood. In the present study, we analyzed a Polish family with X-linked KS. We identified a hemizygous *ANOS1* variant (c.452G > C; p.Cys151Ser) in two affected brothers presenting with hypogonadotropic hypogonadism and olfactory impairment. The identified *ANOS1* variant remains classified as a variant of uncertain significance (VUS); however, its segregation with the KS phenotype and substitution of a highly conserved cysteine residue within the WAP domain supports its potential functional relevance and further investigation. The minigene assay did not reveal detectable effects on *ANOS1* pre-mRNA splicing, while direct transcript analysis was limited by very low *ANOS1* expression in peripheral blood. Whole-blood RNA sequencing revealed significant upregulation of *PROK2* in the affected individuals, and qRT-PCR showed concordant upregulation in both patients. No coding or splice-site variants or detectable exon-usage alterations were identified in *PROK2*. Given the complementary roles of *ANOS1* and *PROK2* in olfactory system development and GnRH neuron migration, and previous evidence linking anosmin-1 to *PK2/PROKR2* signaling, these findings suggest that *ANOS1*-associated dysfunction may be accompanied by secondary changes in functionally related molecular pathways, although this interpretation requires validation in appropriate experimental models.

## 1 Introduction

Kallmann syndrome (KS) is a genetic heterogeneous disorder of the neuroendocrine system characterized by hypogonadotropic hypogonadism and impaired olfactory function, clinically manifested as delayed/absent puberty and infertility, together with anosmia or severe hyposmia (for a review on the subject, see: de Castro et al., 2017; Dodé and Hardelin, 2009; Han et al., 2026). These symptoms reflect developmental defects in two neuronal populations that share ontogenetic origin in the olfactory placode: the olfactory sensory neurons (OSNs) and the gonadotropin-releasing hormone (GnRH) neurons, whose migration up to the hypothalamus is disrupted in KS (Schwanzel-Fukuda and Pfaff, 1989; Teixeira et al., 2010; de Castro et al., 2017). As a consequence, KS patients usually show low levels of circulating gonadotropins (Cariboni and Maggi, 2006). The prevalence of KS is estimated to be approximately 1:8000 in men and 1:40000 in women (Dodé and Hardelin, 2009; de Castro et al., 2017). In addition, various neurological and non-neurological satellite symptoms can occasionally appear (reviewed in de Castro et al., 2017). Increasing evidence suggests that the distinct KS-linked genes participate in shared biological processes, particularly those governing GnRH neuron migration and olfactory system development (Oleari et al., 2021).

Nevertheless, this classical pathophysiological view of KS is too simplistic, as pathogenic variants have been identified in more than 30 genes (de Castro et al., 2017; Stamou and Georgopoulos, 2018). Among them, *ANOS1* was historically the very first gene associated with the X-linked form of the disease and the most studied, although it is implicated in approximately 15% of the total number of KS cases (Kim et al., 2023): *ANOS1* encodes anosmin-1, a large glycoprotein involved in neurogenesis, cell adhesion, axonal guidance, and axon collateral formation and neuronal migration (Rugarli et al., 2002; Soussi-Yanicostas et al., 2002; Cariboni et al., 2004; Bribián et al., 2008; Gianola et al., 2009; Di Schiavi and Andrenacci, 2013; García-González et al., 2016; for reviews, see: de Castro et al., 2014; Díaz-Balzac et al., 2015), and the angiogenesis of the olfactory bulb (Matsushima et al., 2020): all these effects could result in defects in the olfactory circuit and thereby contribute to anosmia. However, the direct role of anosmin-1 in GnRH neuron development remains incompletely understood (Cariboni et al., 2004; Di Schiavi et al., 2022), and mutations in it have been reported in only a subset of clinical cases. In addition, anosmin-1 has been implicated in CNS myelination (Murcia-Belmonte et al., 2016; Villadiego et al., 2023), and we have also suggested that changes in central myelin underlie the olfactory defects in KS (Garcia-Gonzalez et al., 2013). However, despite many studies, KS-associated genes often exhibit variable penetrance and phenotypic heterogeneity, supporting an oligogenic model in which multiple genes and pathways converge to produce the disease phenotype.

Among these, *ANOS1* and *PROK2* have been implicated in complementary aspects of neurodevelopmental regulation of the olfactory–GnRH axis. Experimental and clinical data indicate that both anosmin-1 and PROK2-related pathways contribute to the development of the olfactory–GnRH system, including neuronal migration, axon guidance, and network formation (González-Martínez et al., 2004a; for a recent review, see: Duittoz et al., 2022), suggesting potential convergence at the level of molecular signaling networks regulating neurodevelopment. *PROK2* encodes prokineticin 2, a signaling molecule essential for olfactory bulb development and GnRH neuron migration, and its disruption has been linked to KS phenotypes (Pitteloud et al., 2007; Martin et al., 2011; Kim et al., 2025).

In the present study, we investigated a Polish family with affected male members presenting with classical KS features, including olfactory impairment and hypogonadotropic hypogonadism. We identified a novel hemizygous *ANOS1* variant (c.452G > C; p.Cys151Ser) in affected family members, consistent with X-linked recessive inheritance. Because *ANOS1* expression in peripheral blood was extremely low, assessment of potential splicing abnormalities in patient-derived RNA was not feasible. Therefore, we performed a minigene splicing assay, which did not reveal detectable splicing alterations associated with the c.452G > C variant. To further explore downstream molecular consequences, we performed RNA sequencing, which revealed consistent overexpression of *PROK2* in affected individuals, a finding subsequently validated by qRT-PCR. Notably, no pathogenic variants or splicing alterations in *PROK2* were identified at the DNA or mRNA levels.

Our present findings demonstrate consistent *PROK2* upregulation in the affected individuals and place this observation within the broader concept of convergent molecular pathways underlying KS. By integrating genetic, transcriptomic, and phenotypic data, our study provides a framework for exploring potential relationships between molecular pathways involved in olfactory system formation and GnRH neuronal development. Given the established roles of anosmin-1 in neurogenesis, neuronal migration (including GnRH neurons), axon guidance, collateral formation, and the angiogenesis necessary for olfactory bulb development and maintenance, disruption of these pathways may contribute to both olfactory defects and reproductive dysfunction characteristic of KS (Soussi-Yanicostas et al., 2002; Cariboni et al., 2004; Gianola et al., 2009; García-González et al., 2016; Matsushima et al., 2020; Di Schiavi et al., 2022; Villadiego et al., 2023). Furthermore, these findings underscore the relevance of pathway-level analyses for a more comprehensive understanding of KS pathogenesis, while the proposed relationship between *ANOS1* dysfunction and secondary transcriptional changes should be regarded as a working hypothesis that requires validation in appropriate disease-relevant models.

## 2 Materials and methods

### 2.1 Patients

In this study, we investigated a Polish family spanning three generations, including a 40-year-old mother and her two affected sons, aged 9 and 15 years. Written informed consent was obtained from all participants before inclusion in the study, and the study protocol was approved by the Bioethics Committee at Nicolaus Copernicus University in Toruń, Collegium Medicum in Bydgoszcz (Approval No. KB 371/2023). The two brothers, originating from the same nuclear family, were referred for further clinical and genetic evaluation. Both brothers presented with postnatally diagnosed hypogonadism, manifested by micropenis and cryptorchidism. They also shared several dysmorphic features, including a narrow bitemporal diameter, epicanthal folds, facial asymmetry, and antimongoloid palpebral fissures. In addition, the younger brother had right renal agenesis, a supernumerary nipple, and agenesis of the left maxillary second tooth. Both brothers subsequently experienced delayed puberty. The endocrine evaluation of both brothers demonstrated decreased levels of testosterone, luteinizing hormone (LH), and follicle-stimulating hormone (FSH). Furthermore, both patients exhibited blunted peak responses of LH and FSH following GnRH stimulation, supporting a diagnosis of hypogonadotropic hypogonadism. In addition, both brothers reported anosmia, consistent with involvement of the olfactory system.

Based on the combined clinical, hormonal, and phenotypic findings (see below), the siblings were diagnosed with Kallmann syndrome (KS). The family history, including the presence of unaffected female carriers, was suggestive of X-linked recessive inheritance.

### 2.2 Olfactory assessment

Olfactory function was evaluated using the Odorized Markers Test (Sniffin’ Sticks Screening 12; Burghart Messtechnik, Germany), a standardized, widely used tool for rapid assessment of odor identification (Hummel et al., 1997; Hummel et al., 2001). The test consists of 12 odor-filled pen-like devices, each presented to the participant in a forced-choice paradigm with four possible answers. Participants were asked to identify each odor, and the total score (0–12) was calculated as the number of odors correctly identified.

Individual results were interpreted in relation to normative data adjusted for age and sex, based on the extended population dataset comprising 9,139 subjects (Oleszkiewicz et al., 2019). This approach allowed assessment of odor identification performance relative to age- and sex-adjusted reference values applicable to the Polish population. Because only the 12-item identification test was performed, the results were interpreted as evidence of impaired odor identification rather than as a definitive classification of normosmia, hyposmia, or anosmia, despite a clinical history of self-reported anosmia in both affected individuals.

Olfactory bulb MRI was not available for the affected individuals.

### 2.3 Genetic analysis

Peripheral blood samples were collected from available family members for molecular analyses. For cytogenetic analysis, peripheral blood samples from both affected brothers (III-2 and III-3) were used, and lymphocytes were cultured according to standard procedures. Metaphase chromosomes were analyzed following GTG banding, and karyotypes were described according to the International System for Human Cytogenomic Nomenclature (ISCN 2016). Constitutional karyotyping was performed on both affected brothers, with normal results in each.

Genomic DNA was extracted from peripheral blood leukocytes using standard procedures and a whole blood DNA purification kit (QIAGEN, Germany). Array comparative genomic hybridization (aCGH) was performed in both affected brothers using the commercially available CytoSure Constitutional v3 (8 × 60 K) array (Oxford Gene Technology [OGT], Oxfordshire, UK), according to the manufacturer’s protocol. Genomic copy-number analysis was performed using CytoSure Interpret Software (OGT). No clinically relevant copy-number alterations were identified in either individual.

The entire coding regions of the *ANOS1* gene (exons 1–14 with flanking sequences) and *PROK2* (exons 1–4 with flanking sequences) were amplified and subjected to Sanger sequencing. Sequencing was performed on a 3,130/3130XL Genetic Analyzer (Applied Biosystems) using a BigDye Direct Cycle Sequencing Kit, gene-specific primers (Supplementary Table S1), and a BigDye XTerminator Purification Kit (Thermo Fisher Scientific), according to the manufacturer’s protocols. Variant nomenclature was based on the Ensembl transcript ID ENST00000262648 and described in accordance with Human Genome Variation Society (HGVS) guidelines.

### 2.4 Minigene assay

A minigene splicing assay was performed to assess the effect of the identified variant on mRNA splicing. A genomic fragment of the *ANOS1* gene encompassing intron 3, exon 4, and intron 4 was PCR-amplified from both patient and control DNA using specific primers (Supplementary Table S2). The amplified products were initially cloned into the pGEM-T Easy vector system (Promega, A1380).

Subsequently, the insert was excised with EcoRI (Promega, R6011) and subcloned into the EcoRI-digested, dephosphorylated pCI mammalian expression vector (Promega, E1731). The resulting constructs were transformed into competent cells and verified before downstream applications. HEK293T and SH-SY5Y cells were transiently transfected with the wild-type and mutant (with variant c.452G > C) constructs using jetOPTIMUS reagent (Polyplus) according to the manufacturer’s instructions. Total RNA was extracted, reverse-transcribed into cDNA, and Sanger sequencing was performed to confirm the presence of the *ANOS1* variant in the mutant construct and its absence in the control. Splicing outcomes were analyzed by RT-PCR using specific primers (Supplementary Table S3) and compared between wild-type and mutant constructs.

### 2.5 Assessment of gene expression and splicing patterns

Whole-blood transcriptomic profiling was performed to investigate changes in gene expression in affected family members with KS. Blood sampling was not strictly standardized with respect to time of day, and inflammatory markers or leukocyte subpopulation profiles were not systematically assessed at the time of collection. Total RNA was isolated using TRIzol Reagent according to the manufacturer’s instructions (Thermo Fisher Scientific, 15,596,026), followed by removal of genomic DNA contamination by RapidOut DNA Removal Kit (Thermo Fisher Scientific, K2981). Ribosomal RNA was depleted prior to library preparation using the KAPA RNA HyperPrep Kit with RiboErase.

Paired-end RNA sequencing was carried out on the Illumina NovaSeq X Plus platform, generating approximately 100 million paired-end reads per sample. The study included samples from two affected male siblings, one heterozygous female carrier, and two unaffected controls, enabling comparative transcriptomic analyses across affected, carrier, and control groups.

Quantitative real-time PCR (qRT-PCR) was performed to validate changes in gene expression. Total RNA, previously isolated using TRI Reagent (phenol–chloroform extraction), was reverse-transcribed into cDNA using standard protocols (Thermo Fisher Scientific). qRT-PCR was then carried out using Maxima SYBR Green qPCR Master Mix (Thermo Fisher Scientific, K0251), according to the manufacturer’s instructions. Specific primers (Supplementary Table S4) were used for amplification. Although the standard protocol recommends an annealing temperature of 60 °C, reactions were performed at 51 °C, optimized based on the calculated melting temperatures (Tm) of the primers. Each qRT-PCR reaction was performed in two technical replicates. *HPRT1* and *TBP* were both evaluated as reference genes for normalization. Relative gene expression levels were calculated using the comparative Ct (ΔΔCt) method. Given the limited number of biologically independent samples, no inferential statistical testing was performed on the qRT-PCR data; the analysis was used to assess concordance with the RNA-seq expression pattern. For the principal affected-versus-control comparison, a male control of comparable age was selected.

Additionally, semi-quantitative RT-PCR was performed on cDNA to assess transcript presence using gene-specific primers for *ANOS1, DUSP6*, and *PROK2*. Amplification was carried out using GoTaq Hot Start Green Master Mix (Promega) with 30 PCR cycles, and the resulting products were analyzed to compare splicing patterns between samples.

### 2.6 Bioinformatics analysis and data deposition

The bioinformatic analyses were performed at two levels: (i) differential gene expression analysis and (ii) exon-level expression analysis. All analyses were conducted on the same dataset using different computational tools. Raw sequencing reads were subjected to quality control and filtering using fastp (v 0.23.2). Cleaned reads were aligned to the human reference genome (GRCh38 primary assembly, GENCODE release 49) using HISAT2 (v2.1.1) for gene-level analyses and STAR (v 2.7.11) for exon-level analyses. Gene expression differences were assessed using Cuffdiff (v 2.2.1), with statistical significance defined as an adjusted *p*-value (*q*-value) ≤ 0.05. Multiple pairwise and grouped comparisons were performed between affected individuals, one carrier, and controls.

Exon-level differential expression and alternative splicing analyses were conducted using the DEXSeq R/Bioconductor package (v. 1.54.1). Exon-level annotations and read counts were generated using the accompanying DEXSeq scripts dexseq_prepare_annotation.py and dexseq_count.py, respectively. Significant differences in exon usage were defined as adjusted *p*-values < 0.05. Additional expression quantification was performed using TPMCalculator (v 0.0.5) to estimate transcript abundance (TPM), particularly for selected genes of interest. Downstream analyses focused on key KS-related and neurodevelopmental genes, including *ANOS1, CCDC141, DUSP6, PROK2, SMCHD1*, and *TCF25*.

All raw and processed RNA-seq data have been deposited in the Gene Expression Omnibus (GEO) database under accession number GSE318901.

### 2.7 *In silico* analysis of *ANOS1* variant and *PROK2*-related pathways

The potential functional impact of the identified *ANOS1* variant was assessed using multiple *in silico* prediction tools, including SIFT, PolyPhen-2, MutationTaster, and REVEL. These algorithms evaluate the effects of sequence variants using evolutionary conservation, predicted structural changes, and integrated annotation data.

Additional variant interpretation was performed using Alamut Visual Plus v. 1.7.2 (SOPHiA GENETICS, Switzerland), Franklin (Genoox), and GeneBe. These platforms integrate population, computational, functional, and clinical evidence and support variant classification in accordance with the American College of Medical Genetics and Genomics/Association for Molecular Pathology (ACMG/AMP) guidelines (Richards et al., 2015).

To explore possible relationships between *ANOS1* and *PROK2*, gene interaction and pathway analyses were conducted using the publicly available STRING database. These tools were used to identify shared interaction networks, co-expression patterns, and involvement in common biological pathways.

The presence and frequency of the identified variants in control populations were evaluated using dbSNP and the Genome Aggregation Database (gnomAD). The *ANOS1* c.452G > C (p.Cys151Ser) variant was absent from gnomAD despite adequate sequencing coverage of the corresponding genomic position, as assessed using population frequency data aggregated by VarSome.

## 3 Results

### 3.1 Clinical and genetic findings

Sanger sequencing was performed in selected family members to investigate the genetic basis of KS in the studied pedigree. The family structure was consistent with an X-linked recessive inheritance pattern. Two affected brothers (III-2 and III-3) were found to be hemizygous for a novel missense variant in the *ANOS1* gene (c.452G > C; p.Cys151Ser). Their mother (II-2) and maternal grandmother (I-2) were identified as heterozygous carriers, whereas the father was not a carrier (Figure 1). Additionally, the maternal aunt (II-1) and her son (III-1), a cousin of the affected siblings, were also found to carry the same variant, further supporting segregation of the variant within the maternal lineage.

**Figure 1:**
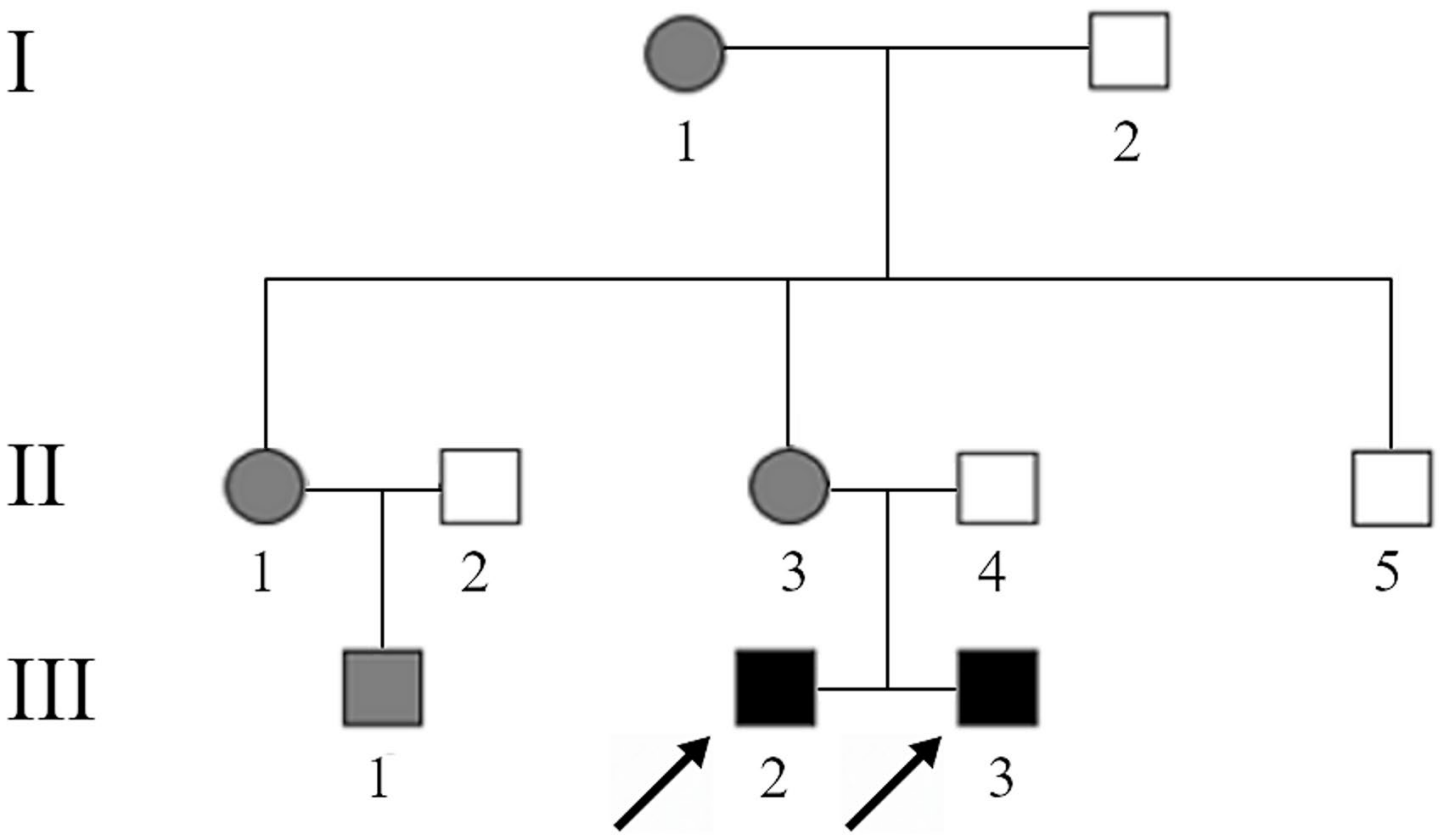
Family pedigree. Squares indicate males and circles indicate females. Black-filled symbols represent individuals diagnosed with Kallmann syndrome, whereas gray-filled symbols indicate carriers of the *ANOS1* variant without a clinical diagnosis of KS. Open symbols denote individuals without the variant. Arrows indicate the probands.

Sanger sequencing chromatograms confirmed hemizygosity for the *ANOS1* variant in the affected brothers (III-2 and III-3) and heterozygosity in their mother (Figure 2). Constitutional karyotyping and aCGH analyses yielded normal results in both affected brothers.

**Figure 2:**
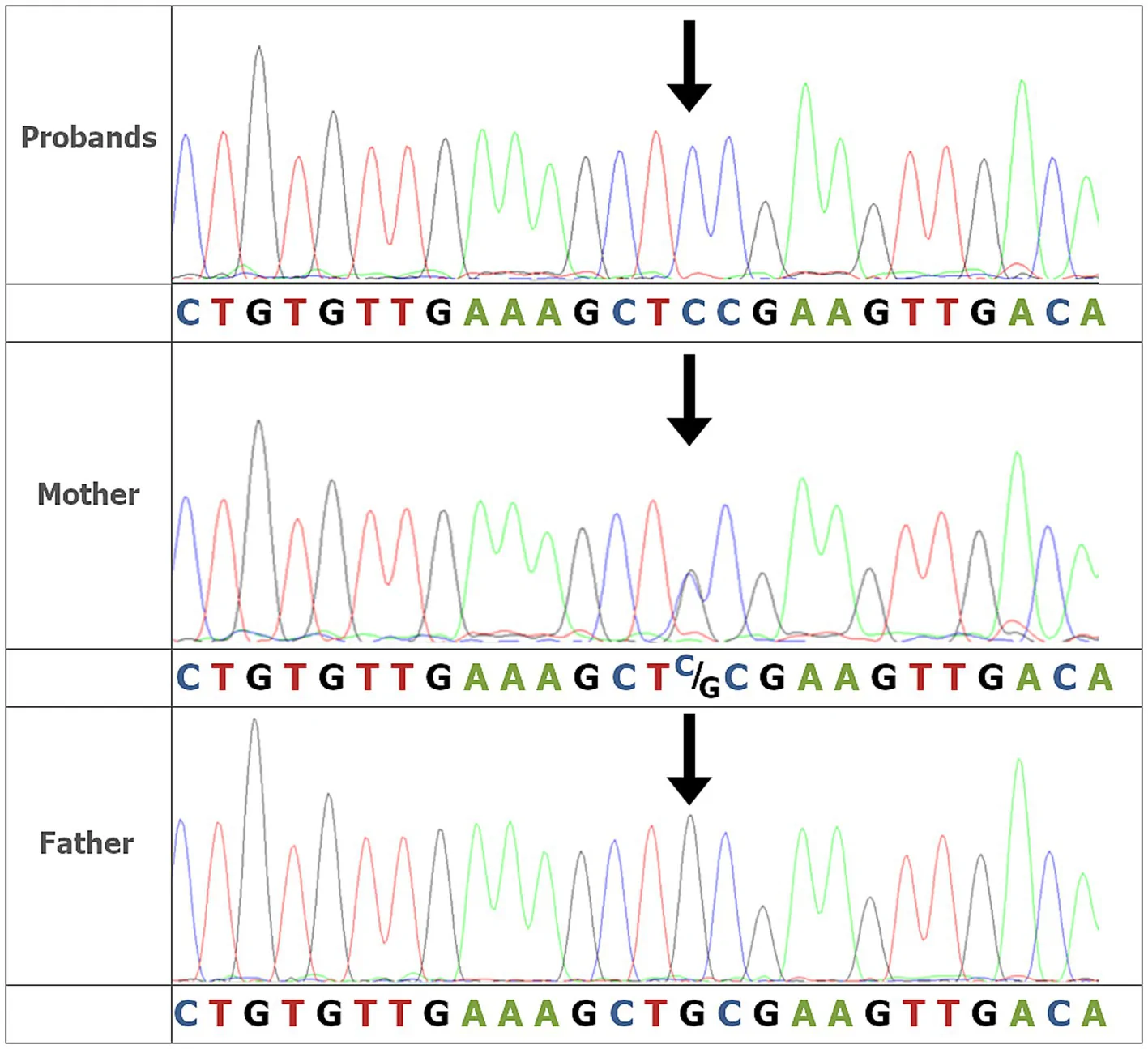
Sanger sequencing results of the analyzed family. Partial chromatograms showing the variant site in the *ANOS1* gene. The black arrows indicate the position of the c.452G > C variant.

The identified *ANOS1* variant has not been previously reported in public databases, including ClinVar, dbSNP, and gnomAD. *In silico* prediction tools, including SIFT and PolyPhen-2, suggested a potentially deleterious effect of the variant on protein function. However, integrated variant interpretation platforms, including VarSome and Franklin, classified the variant as a variant of uncertain significance (VUS) based on available ACMG/AMP evidence (Supplementary Table S5).

Following genetic analysis, additional experimental analyses were undertaken to further investigate the potential consequences of the variant, as described below. The novel variant has been submitted to the Leiden Open Variation Database (LOVD) under accession number #0000931586.

Olfactory function was assessed using the Sniffin’ Sticks test (12 odor-identification markers), which revealed markedly impaired odor identification in both affected brothers. Individual III-2 correctly identified 4 odors for the left nostril, 4 for the right nostril, and 4 with bilateral nostril testing. Individual III-3 identified 4 odors for the left nostril, 6 for the right nostril, and 4 with bilateral testing. In contrast, their mother (II-2) demonstrated odor identification performance within the normal range (Supplementary Table S6).

### 3.2 Structural context of the *ANOS1* variant

The *ANOS1* gene, located on chromosome Xp22.3, consists of 14 exons spanning approximately 220 kb of genomic DNA and encodes a 680-amino-acid protein, anosmin-1. This secreted glycoprotein exhibits a multidomain organization, including an N-terminal signal peptide, a cysteine-rich region, a whey acidic protein (WAP)-like domain, four fibronectin type III (FnIII) repeats, and a C-terminal histidine-rich region (Figure 3; Murcia-Belmonte et al., 2010; Murcia-Belmonte et al., 2014). The identified variant (p.Cys151Ser) is located within exon 4 and corresponds to the WAP domain of anosmin-1. This domain is characterized by a conserved four-disulfide core motif formed by eight cysteine residues (Cys134, Cys147, Cys151, Cys157, Cys163, Cys164, Cys168, and Cys172), which are critical for proper folding and structural integrity. Sequence alignment across species demonstrates a high degree of evolutionary conservation of these cysteine residues within the WAP domain (Figure 4), supporting their functional relevance. Although no direct functional evidence indicates that the c.452G > C (p.Cys151Ser) variant disrupts WAP domain function, its location within a highly conserved cysteine residue suggests that it may influence the structural stability of this domain. Given that the WAP domain has been implicated in mediating protein–protein interactions in the extracellular environment (Smith, 2011; Garcia-Gonzalez et al., 2013), any structural alteration in this region could hypothetically affect anosmin-1 interactions with its molecular partners.

**Figure 3:**
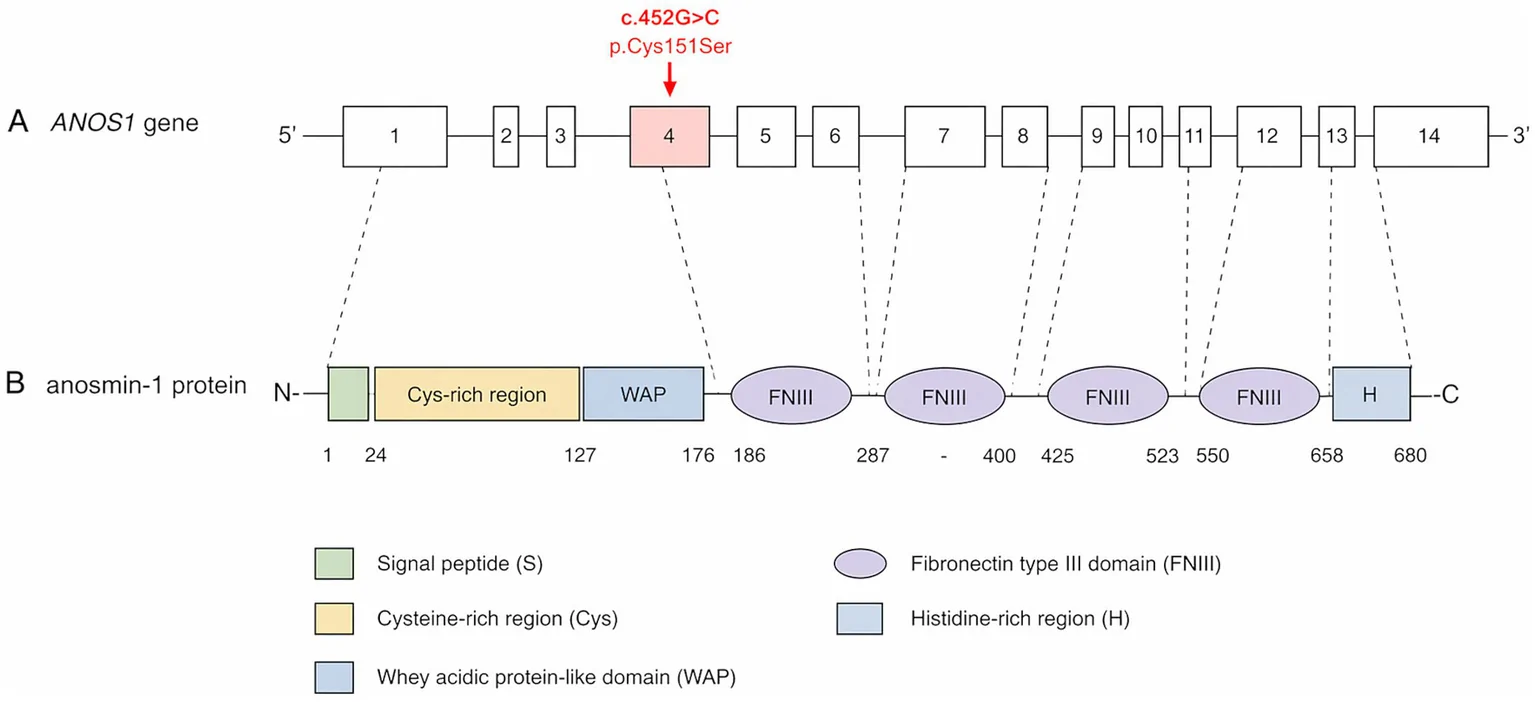
Structure of the *ANOS1* gene and anosmin-1 protein. **(A)** Organization of the *ANOS1* gene. Coding exons are represented by boxes and introns by connecting lines. The location of the identified variant (c.452G > C; p.Cys151Ser) in exon 4 is indicated. **(B)** Domain structure of the anosmin-1 protein, including the signal peptide (S), cysteine-rich region (Cys), whey acidic protein-like domain (WAP), fibronectin type III domains (FNIII), and histidine-rich region (H).

**Figure 4:**
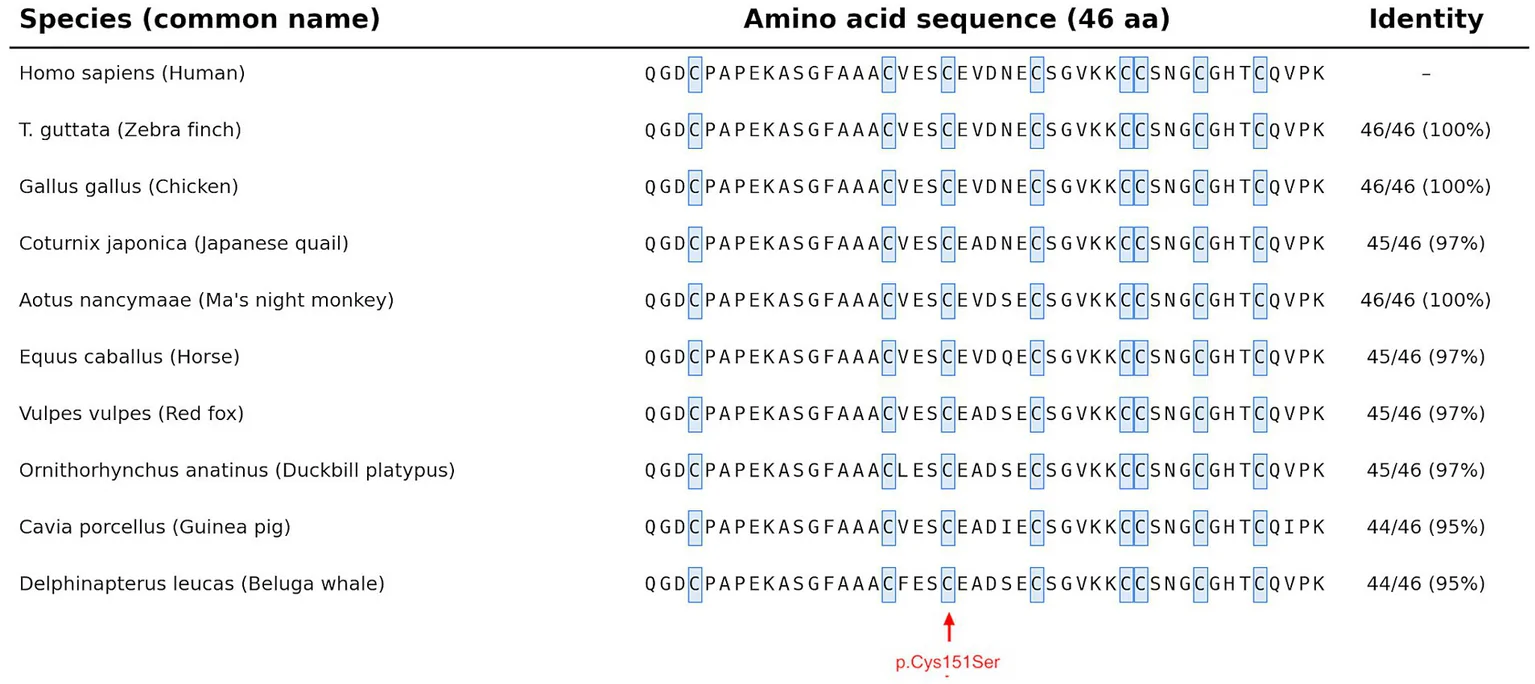
Comparison of amino acid sequences of the WAP domain (46 aa) among selected vertebrate species. The human (*Homo sapiens*) sequence (UniProt/Swiss-Prot accession P23352) is used as the reference. The cysteine residue corresponding to human Cys151 is conserved in all species and highlighted in blue. Percentage identity is shown as matches/total amino acids.

### 3.3 Minigene assay

A schematic representation of the *ANOS1* minigene construct used to analyze the effect of the c.452G > C variant on pre-mRNA splicing is shown in Figure 5A. Because *ANOS1* expression in peripheral blood is negligible, direct analysis using patient-derived RNA was not feasible; therefore, a minigene assay was employed. A genomic fragment encompassing intron 3, exon 4, and intron 4 was amplified from patient DNA and initially cloned into the pGEM-T Easy vector system for propagation. Subsequently, the insert was subcloned into the pCI mammalian expression vector to generate minigene constructs for splicing analysis.

**Figure 5:**
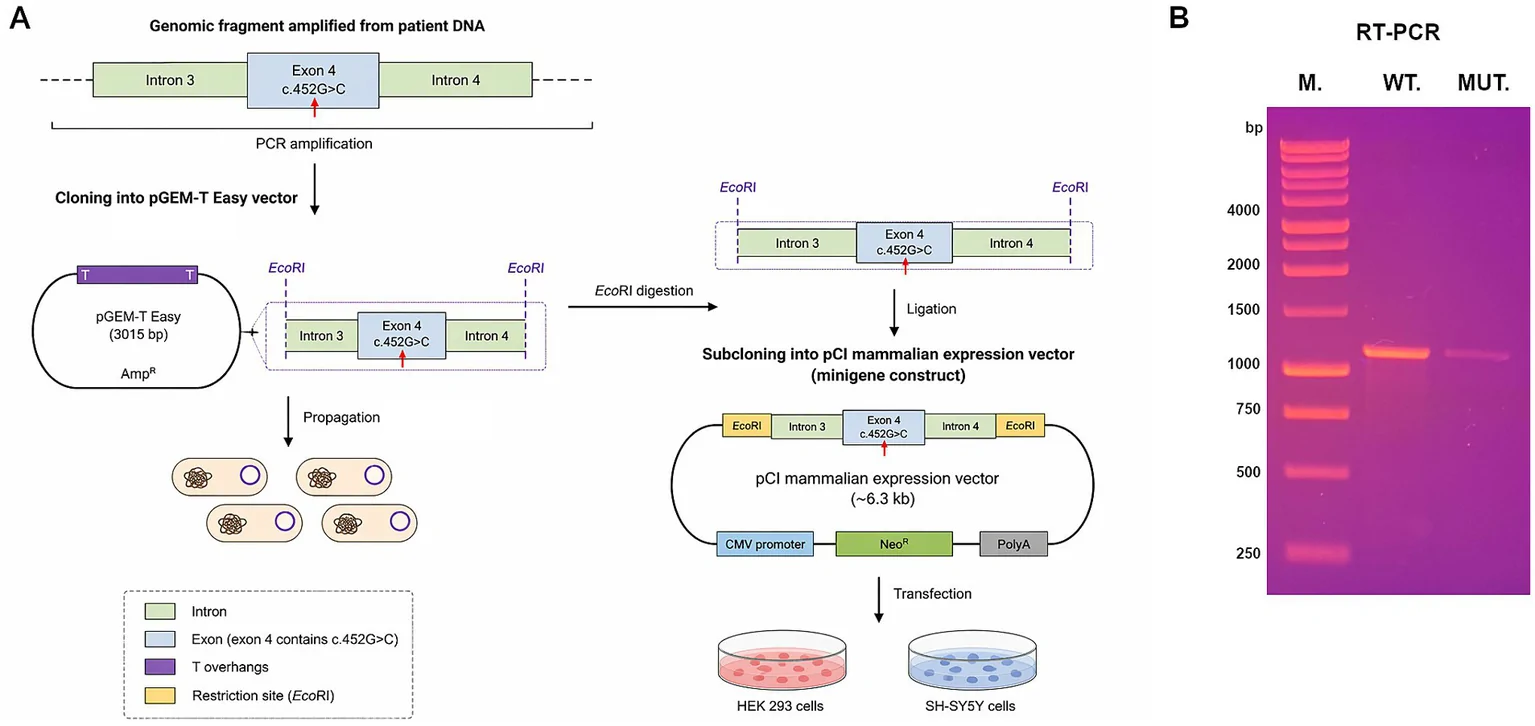
Minigene analysis of the *ANOS1* c.452G > C (p.Cys151Ser) variant shows no detectable splicing alterations. **(A)** Schematic representation of the *ANOS1* minigene construct containing intron 3, exon 4, and intron 4, generated from patient DNA and cloned into the pCI expression vector. **(B)** RT-PCR analysis of transcripts derived from wild-type (WT) and mutant (MUT) constructs in SH-SY5Y cells showing a single product of the expected size (1,130 bp), indicating no detectable splicing alterations associated with the c.452G > C variant.

The constructs were transfected into human HEK293T and SH-SY5Y cell lines. RT-PCR analysis of cDNA synthesized from total RNA extracted from transfected cells revealed a single product of the expected size (1,130 bp) for both wild-type and mutant constructs, indicating that the c.452G > C variant did not cause detectable alterations in *ANOS1* pre-mRNA splicing under the experimental conditions used (Figure 5B).

### 3.4 Transcriptomic profiling identifies gene-specific expression changes with emphasis on *PROK2* dysregulation

Genome-wide transcriptomic analysis of whole-blood RNA-seq data did not reveal widespread gene-level alterations across the analyzed comparisons (adjusted *p*-value <0.05; GEO: GSE318901). Among the selected candidate genes, most—including *ANOS1, CCDC141, DUSP6, SMCHD1*, and *TCF25*—did not show statistically significant differential expression between affected individuals and controls (Supplementary Table S7). Notably, *ANOS1* expression levels were consistently low across all samples (TPM < 0.02), precluding reliable comparative analysis at both gene and exon levels. This observation was further supported by qRT-PCR, which showed that *ANOS1* was not detectable in peripheral blood.

In contrast, *PROK2* exhibited significant differential expression in the RNA-seq analysis. Specifically, a combined analysis of both affected individuals compared with the male control revealed statistically significant upregulation (FPKM: affected, 66.4 vs. control, 34.28; FDR-adjusted *p*-value = 0.00853), corresponding to an approximately 1.94-fold increase in expression. No significant exon-level differences were detected for *PROK2* (Supplementary Figure S1). The figure was generated using the plotDEXSeq function from the DEXSeq package; exons or exonic parts showing significant differential usage would be highlighted, but none were identified. These findings suggest that the transcriptional upregulation of *PROK2* occurs without detectable alterations in exon usage.

### 3.5 Exon-level analysis identifies differential exon usage in *DUSP6* and *TCF25*

*DUSP6* encodes an ERK1/2-specific dual-specificity phosphatase that functions as a negative-feedback regulator of FGF/FGFR signaling, a pathway with an established role in developmental processes relevant to KS (Li et al., 2007). Although no global gene-level changes were observed for *DUSP6*, exon-level analysis identified a significant alteration in exon 3 (exonic_part E001; adjusted *p* = 8.82 × 10^−10^), corresponding to a reduction in exon usage (~2.3-fold decrease). Similarly, differential exon usage was detected in *TCF25*, involving multiple exonic regions with moderate fold changes. However, these exon-level differences were not accompanied by consistent changes in overall gene expression and were not further supported by downstream validation.

### 3.6 qRT-PCR validation concordant *PROK2* upregulation but not *DUSP6* alterations

To validate RNA-seq findings, qRT-PCR analysis was performed for *PROK2* and *DUSP6* (Figure 6A, Supplementary Table S8). qRT-PCR showed increased *PROK2* expression in both affected individuals compared with control samples. Patient 1 (corresponding to K3_KA) showed approximately 1.65-fold upregulation, while Patient 2 (K1_KN) exhibited an approximately 2.98-fold increase in expression, consistent with the RNA-seq results.

**Figure 6:**
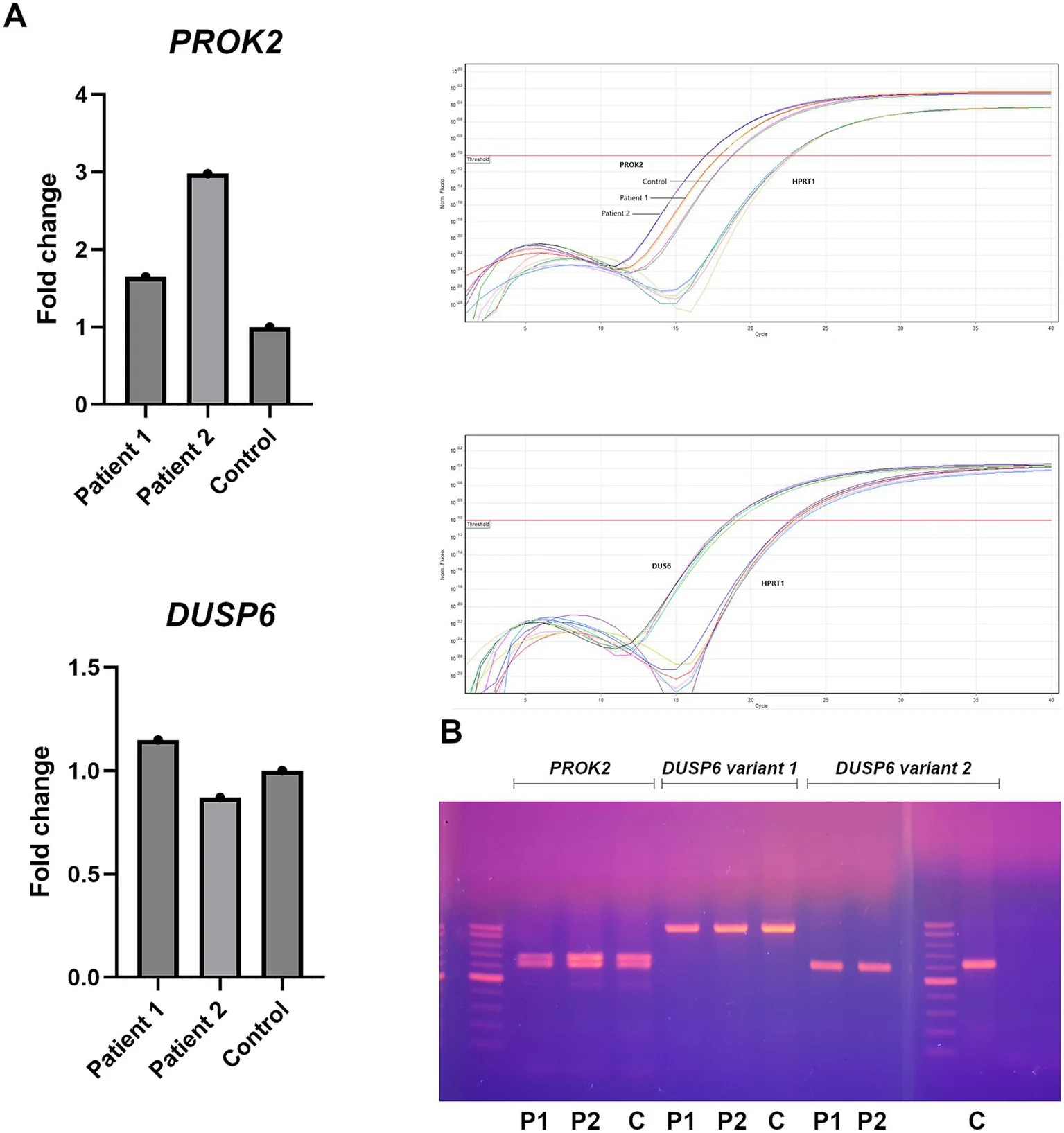
Validation of *PROK2* and *DUSP6* expression and splicing. **(A)** qRT-PCR shows concordant upregulation of *PROK2* in both patients, whereas *DUSP6* shows no consistent change. **(B)** Semi-quantitative RT-PCR reveals no splicing differences in either gene between patients and controls.

In contrast, qRT-PCR analysis of *DUSP6* did not confirm differential expression, with comparable expression levels observed across both patients and controls, indicating that RNA-seq–detected exon-level differences do not translate into detectable gene-level expression changes.

### 3.7 No evidence of splicing abnormalities in *PROK2* and *DUSP6*

Semi-quantitative RT-PCR analysis did not reveal any differences in transcript size or pattern for either *PROK2* or *DUSP6* between affected individuals and controls (Figure 6B), indicating the absence of major splicing defects in these genes.

### 3.8 Sanger sequencing excludes coding variants in *PROK2*

To determine whether the observed *PROK2* upregulation could be attributed to underlying sequence variation, Sanger sequencing of the *PROK2* gene was performed in both affected individuals. The analysis covered the reference sequence NG_008275.1, which encompasses the *PROK2* transcript NM_001126128.1. No sequence variants were identified in either proband compared to the reference sequence, indicating that the increased *PROK2* expression is not associated with detectable coding or splice-site variants (Sanger sequencing data not shown; exon-usage analysis shown in Supplementary Figure S1).

### 3.9 Interaction network analysis of *ANOS1* and *PROK2*

To explore potential relationships between *ANOS1* and *PROK2*, gene interaction and pathway analysis were performed using the STRING database (Figure 7). Although no direct interaction between these genes was identified, both were found within a shared network involving key regulators of olfactory development and GnRH neuron migration, including *CHD7, FGFR1, FGF8*, and *PROKR2* (Cho et al., 2019). These results place *ANOS1* and *PROK2* within a shared network involving common neurodevelopmental regulators.

**Figure 7:**
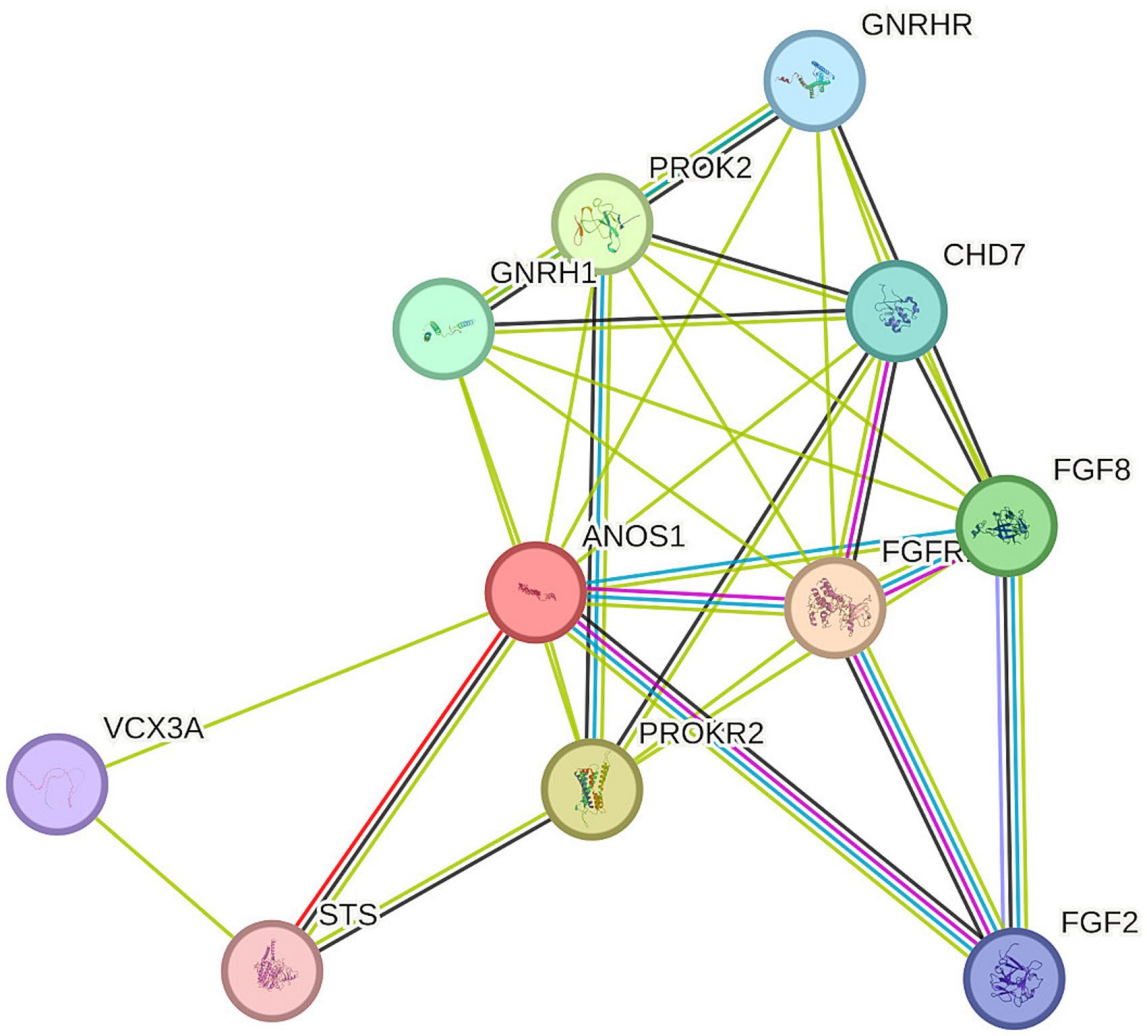
STRING network showing functional associations between *ANOS1* and *PROK2*. *ANOS1* and *PROK2* are indirectly connected through shared regulators of olfactory and GnRH neuron development, including *FGFR1, FGF8, CHD7*, and *PROKR2*.

## 4 Discussion

Our study provides evidence that the c.452G > C variation in *ANOS1* may be associated with secondary transcriptional dysregulation of *PROK2*, supporting the notion that convergent molecular pathways underlie KS. While *ANOS1* has long been recognized as a key determinant of X-linked KS (Dodé and Hardelin, 2009; de Castro et al., 2017), the downstream consequences of its disruption remain insufficiently characterized. Our integrated genetic and transcriptomic analyses show that, despite the absence of detectable effects on *ANOS1* splicing and the very low *ANOS1* expression in peripheral blood, *PROK2* is consistently upregulated in the affected individuals.

A central observation of this study is the relationship between the primary genetic finding and measurable transcriptional output. The identified *ANOS1* variant (p.Cys151Ser), located within the highly conserved WAP domain, does not alter mRNA splicing, as confirmed by the minigene assay. However, its position within a structurally critical cysteine motif suggests a potential impact on protein folding or stability. The WAP domain is characterized by a conserved eight-cysteine framework forming four disulfide bonds, which are essential for maintaining its tertiary structure (Esteban et al., 2013; Robertson et al., 2001; Murcia-Belmonte et al., 2014). Substitutions affecting these cysteine residues have been previously shown to disrupt disulfide bond formation and impair protein stability: for example, the p.Cys163Tyr and p.Cys172Arg variants identified in KS patients are predicted to disrupt the Cys151-Cys163 and Cys157-Cys172 disulfide bonds, respectively, leading to misfolding of the WAP domain (Oliveira et al., 2001; Sato et al., 2004; Nie et al., 2017). Given this structural context, the p.Cys151Ser substitution described in our present study may have a similar effect, potentially destabilizing the disulfide bond network and altering the 3D conformation of anosmin-1. The WAP domain is known to mediate protein–protein interactions in extracellular signaling environments (Ranganathan et al., 1999; Esteban et al., 2013; Murcia-Belmonte et al., 2014), and the disruption of its disulfide-bond architecture may impair interactions of anosmin-1 with its molecular partners (Murcia-Belmonte et al., 2022; Hu et al., 2009). This is consistent with previous studies demonstrating that anosmin-1 functions as a modulator of extracellular signaling cues essential for neural development, including interactions with FGFR1 and heparan sulfate proteoglycans (González-Martínez et al., 2004b; Murcia-Belmonte et al., 2010; Kim et al., 2008; Bribián et al., 2020). Beyond its established role in neuronal migration, anosmin-1 has also been implicated in axon guidance, collateral formation, neurogenesis, and progenitor cell proliferation (Soussi-Yanicostas et al., 2002; Gianola et al., 2009; García-González et al., 2016; Villadiego et al., 2023). Therefore, defects associated with *ANOS1* variants may arise not only from impaired neuronal migration but also from abnormalities in neuronal development and connectivity within the olfactory–GnRH system. Furthermore, alterations in residues adjacent to heparan sulfate (HS)-binding regions have been shown to influence anosmin-1 affinity for extracellular matrix components, potentially affecting its mobility and signaling capacity (Kim et al., 2008). Finally, anosmin-1-linked modifications in myelination of the lateral olfactory tract would result in olfactory defects (Garcia-Gonzalez et al., 2013; Murcia-Belmonte et al., 2016). Taken together, these observations support the hypothesis that the identified variant may affect protein function despite the absence of detectable changes in mRNA processing.

We want to highlight that our present RNA-seq and qRT-PCR analyses revealed a consistent increase in *PROK2* expression in both affected individuals. This finding is particularly notable because we did not see coding or splice-site variants in *PROK2*, nor any evidence of alternative splicing. These findings indicate that the observed *PROK2* difference occurs at the expression level rather than reflecting a primary *PROK2* coding or splice-site defect. Because blood sampling was not standardized across all potentially relevant variables, contributions from circadian phase, inflammatory status, leukocyte composition, pubertal status, and treatment cannot be excluded. Moreover, because the two affected individuals are siblings, they cannot be considered fully independent biological replicates. These factors should therefore be considered when interpreting the observed difference in *PROK2* expression. If additional patients carrying the same or other *ANOS1* variants become available, replication of the transcriptomic and qRT-PCR findings in an independent cohort would provide an important opportunity to assess the reproducibility of this observation, although such validation may be challenging given the rarity of *ANOS1*-associated KS.

The biological relevance of this latter observation is supported by the known roles of *ANOS1* and *PROK2* in overlapping neurodevelopmental processes. *PROK2* encodes prokineticin-2, a secreted signaling molecule that plays a role in olfactory bulb morphogenesis and GnRH neuron migration (Ng et al., 2005). Loss-of-function variants in *PROK2* and its receptor *PROKR2* have been independently associated with KS (Dodé et al., 2006). Although these genes have traditionally been considered part of distinct genetic pathways, growing evidence suggests functional convergence in the development of the olfactory system and in neuroendocrine regulation. Our findings support a potential functional link between *ANOS1* dysfunction and *PROK2* dysregulation, thereby connecting two canonical KS genes within a shared regulatory framework. In this sense, a possible explanation for *PROK2* upregulation in our KS patients would be the activation of compensatory mechanisms in response to the impaired extracellular microenvironment triggered by anosmin-1 dysfunction, as, for example, has been suggested for cues guiding GnRH neurons migration (Cariboni et al., 2007). Alternatively, *ANOS1* disruption may indirectly affect transcriptional networks regulating neurodevelopmental pathways, thereby altering *PROK2* expression. Both hypotheses are consistent with recent evidence indicating that disruptions of distinct KS-associated genes may converge on shared molecular and transcriptional pathways (Moon and Zhao, 2022). Although DEXSeq identified altered exon usage in *DUSP6*, this finding was not supported by qRT-PCR at the gene-expression level or by semi-quantitative RT-PCR at the transcript-pattern level, suggesting that it may represent a subtle regulatory difference or a technical artifact. Altogether, the consistent upregulation of *PROK2* in both transcriptomic and qRT-PCR analyses supports its potential role as a secondary transcriptional response in *ANOS1*-associated KS.

In addition to the small cohort size, another limitation of our study is the use of peripheral blood as the RNA source. *ANOS1* expression in whole blood is very low in publicly available transcriptomic datasets, including the Human Protein Atlas, consistent with TPM values below 0.02 observed in our RNA-seq data (Uhlén et al., 2015). Consequently, our analyses cannot directly capture the molecular consequences of *ANOS1* dysfunction in disease-relevant tissues, particularly within the developing olfactory system and the hypothalamic–GnRH neuronal axis. Nevertheless, the consistent dysregulation of *PROK2* in blood transcriptomes suggests that systemic transcriptional signatures associated with KS may be detectable outside the primary affected tissues. Future studies using neuronal models, induced pluripotent stem cell-derived cells, or olfactory-derived cellular models will be required to validate these findings in a more physiologically relevant context.

Taken together, our present results are consistent with a model in which *ANOS1*-associated molecular alterations may not act in isolation but could perturb broader transcriptional networks involved in olfactory and GnRH neuron development. The consistent upregulation of *PROK2* in both affected individuals may represent a potential point of convergence between molecular pathways implicated in KS. This interpretation is consistent with evidence that KS-associated genes, despite their diverse molecular functions, may converge on shared biological processes. For example, disruption of *HS6ST1* and *FGFR1* results in highly overlapping transcriptomic changes affecting common pathways, including extracellular signaling and chromatin regulation (Moon and Zhao, 2022). Moon and Zhao further proposed that these effects may involve shared regulatory nodes, including the transcription factors *SOX9/SOX10* and the chromatin regulator *CHD7*, supporting coordinated pathway-level regulation in KS. We therefore propose, as a working hypothesis, that impaired anosmin-1 function may be associated with secondary transcriptional dysregulation of *PROK2* and other genes involved in olfactory and neuroendocrine development through shared extracellular or intracellular signaling networks. Although this hypothesis requires validation in disease-relevant cellular models, it provides a plausible framework linking a primary genetic finding to downstream molecular alterations in KS.

## Data Availability

The datasets presented in this study can be found in online repositories. The names of the repository/repositories and accession number(s) can be found at: https://www.ncbi.nlm.nih.gov/geo/, GSE318901.
