# Supplementary material for: ANOS1 variant and PROK2 upregulation in familial Kallmann syndrome: integrative genetic, transcriptomic, and phenotypic study
Source: Front Mol Neurosci. 2026 Oct 22;19:1960672. doi: 10.3389/fnmol.2026.1960672 (PMC13736604; doi:10.3389/fnmol.2026.1960672)
Supplement: Supplementary file 1 [file Data_Sheet_1.DOCX]

| **Primer R 5’-3’** | **Primer F 5’-3’** | **Product [bp]** | **Number of exons** | **Gene** |
| --- | --- | --- | --- | --- |
| GTCTCCAGATGCGTCCATGA | GAACATTTGCCAGGGTCTCG | 331 | 14 | *ANOS1* |
| GAAAGGGAGAAAAGCGGGGA | CCATATGGCAGAGAACCCCC | 589 | 13 | *ANOS1* |
| AGCCACCTCTGAAGGTCAGT | TCCAGTCGCCTAATCCTGGT | 338 | 12 | *ANOS1* |
| CACGGTCTTGTGAAACGCAG | GGAAAACATCCTTGTTGGATGGA | 366 | 11 | *ANOS1* |
| CCATTCTGCTTTCCACTTCCA | AAGTTCAAGAACCCCAGGCT | 312 | 10 | *ANOS1* |
| ACTGTGGCTTGACATTTACTTCT | GCCATCTTGCCCAGGAATCT | 304 | 9 | *ANOS1* |
| TGCCTTGTTGTGATATGGCG | CGTGGAAGGTTTGTAACGCA | 258 | 8 | *ANOS1* |
| TCCTGCATCCATGGCATCTC | AGCAATGCTTCACGTGTTGA | 411 | 7 | *ANOS1* |
| CAGCAAAGCCACCTGTTGAC | TGTTCCCTGTGCTCTGTGAC | 280 | 6 | *ANOS1* |
| GCGTAGCTATGTAGCAGACACT | ACTTCAATTTGAGTCACACGCA | 300 | 5 | *ANOS1* |
| CCTTCCCTAGGCACACACAG | CAAACAAAGGTTTGGTGGGGA | 401 | 4 | *ANOS1* |
| CCATGACCCCACGTAAGCAT | AGAGCAGCTGATTCTTTGGTTT | 270 | 3 | *ANOS1* |
| CTGGTCTAACCAGAGGGCAT | TGTGTATGTGTAGCTTTCTAATGGA | 447 | 2 | *ANOS1* |
| CCCCGACTGTAAGATCCACG | AGGATTGAACTTTCCGGCTCA | 481 | 1 | *ANOS1* |
| GCAAGAGCATTTCTTTCTGGCA | GCCTAATGGGTAGTTAACGCTC | 334 | 4 | *PROK2* |
| GGGGCTGAACTGATAGGACA | CTTGCTCCGCCAGTT | 269 | 3 | *PROK2* |
| TTTGTCGAGCACGTTACCCA | TCCTTAGCCTGAGTCTTTCCTG | 280 | 2 | *PROK2* |
| GCTCTAGCCTGCCCTTCA | CACGTGCTCGTCTGGGA | 450 | 1 | *PROK2* |

**Table S1. Primers used for Sanger sequencing of *ANOS1* and *PROK2*.** Primer sequences were designed to amplify the coding regions and flanking sequences of the *Homo sapiens ANOS1* gene (RefSeqGene, chromosome X; NCBI Reference Sequence: NG_007088.2) and the *PROK2* gene (NCBI Reference Sequence: NG_008275.1; transcript NM_001126128.1).

**Table S2. Primers used for genomic PCR amplification.** The listed primer sequences were used to amplify the *ANOS1* gene fragment (NM_000216) for the minigene splicing assay.

| **Primer R 5’-3’** | **Primer F 5’-3’** | **Product [bp]** | **Gene** |
| --- | --- | --- | --- |
| GAATGACTTGCTCTGCCCCA | GGGCCCTCAAATTGTACTGAAC | 775 | *ANOS1* |

**Table S3. Primers used for RT-PCR analysis.** The listed primer sequences were used to assess splicing outcomes of *ANOS1* transcripts in the minigene assay.

| **pCI-ANOS1-R** | **pCI-ANOS1-F** | **Product [bp]** | **Gene** |
| --- | --- | --- | --- |
| TCCCCCTGAACCTGAAACAT | TTGCGTTTCTGAAGGCACCTA | 1130 | *ANOS1* |

**Table S4. Primers used for qRT-PCR analysis.** Primer pairs were used to quantify the expression of *ANOS1* (NM_000216), *PROK2* (NM_021935), and *DUSP6* (NM_001946). *HPRT1* (NM_000194) and *TBP* (NM_003194) served as reference genes for normalization.

| **Primer R 5’-3’** | **Primer F 5’-3’** | **Product [bp]** | **Gene** |
| --- | --- | --- | --- |
| AGCGGAGAAAGACTACGGATGG | GGACACCTTTGCACTCTTCAGC | 134 | *ANOS1* |
| GCTGCCATCCACTGACTCGTAA | CTCCAGAGCGATTACTTTTGGGC | 202 | *PROK2* |
| CTCGGATCACTGGAGCCAAAAC | GTCACAGTGACTGAGCGGCTAA | 132 | *DUSP6* |
| CATTATGCTGAGGATTTGGAAAGG | CTTGAGCACACAGAGGGCTACA | 129 | *HPRT1* |
| TGTATCCACAGTGAATCTTGGTTG | GGTTCGTGGCTCTCTTATCCTC | 124 | *TBP* |

**Table S5. ACMG/AMP evidence assessment of the *ANOS1* c.452G>C (p.Cys151Ser) variant.** Evidence was evaluated according to the ACMG/AMP framework (Richards et al., 2015), with consideration of subsequent ClinGen Sequence Variant Interpretation (SVI) recommendations. The variant remains classified as a variant of uncertain significance (VUS). The negative minigene assay was not considered functional evidence for or against the pathogenicity of the p.Cys151Ser substitution because the assay specifically evaluates pre-mRNA splicing and does not assess anosmin-1 protein folding, stability, secretion, or molecular interactions. Constitutional karyotyping and aCGH did not identify chromosomal abnormalities or clinically relevant copy-number alterations in either affected brother; these findings were considered supportive of the clinical context but were not assigned a separate ACMG/AMP criterion.

| **ACMG/AMP criterion** | **Evidence for ANOS1 c.452G>C (p.Cys151Ser)** | **Application** | **Interpretation** |
| --- | --- | --- | --- |
| PM1 | p.Cys151Ser affects one of the highly conserved cysteine residues forming the four-disulfide core of the anosmin-1 WAP domain. Previous functional studies have demonstrated the importance of the WAP domain for anosmin-1 activity and modulation of *PK2/PROKR2* signaling. | PM1_Supporting | Supporting evidence. The location is functionally relevant; however, in the absence of *ANOS1*-specific ACMG/AMP specifications demonstrating that this region meets all requirements of a mutational hotspot/critical domain without benign variation, the criterion was conservatively downgraded to supporting strength. |
| PM2 | The variant is absent from population databases, including gnomAD. | PM2_Supporting | Supporting evidence. In accordance with ClinGen SVI recommendations, absence/rarity in population databases was applied at supporting rather than moderate strength. |
| PM5 | A different substitution affecting the same amino acid residue, p.Cys151Trp, was previously reported in a cohort of patients with KS (Nie et al., 2017). | Not applied | p.Cys151Trp was reported as a novel variant predicted to be pathogenic, but it has not been independently established as pathogenic/likely pathogenic to the level required for PM5. |
| PP1 | The variant was identified in both affected brothers and was inherited through the maternal lineage; it was also identified in hemizygous form in the maternal cousin III-1, whose KS phenotype could not be independently and unequivocally assessed in the present study. | PP1 | Supporting evidence for co-segregation, the phenotype of III-1 was not used as definitive evidence of segregation. |
| PP3 | *In silico* analyses, including SIFT and PolyPhen-2, support a deleterious effect of the p.Cys151Ser substitution, and Cys151 is evolutionarily conserved. | PP3 | Supporting computational evidence; not used at an increased strength |
| PS3 | No direct functional assay assessing the effect of p.Cys151Ser on anosmin-1 protein function was performed. | Not met | The minigene assay evaluates pre-mRNA splicing and cannot establish a damaging effect of the missense substitution on anosmin-1 protein function. |
| BS3 | The minigene assay showed no detectable alteration of *ANOS1* pre-mRNA splicing. | Not met | Normal splicing in the minigene system does not demonstrate normal folding, stability, secretion, or molecular interactions of the p.Cys151Ser anosmin-1 protein. |
| PP4 | The phenotype and X-linked inheritance pattern are highly compatible with *ANOS1*-associated KS; however, KS is genetically heterogeneous. | Not applied | The phenotype cannot be considered specific to a single genetic etiology, particularly given the large number of genes associated with KS/CHH. |
| **Overall classification** | **PM1_Supporting + PM2_Supporting + PP1 + PP3** | **Variant of uncertain significance (VUS)** | The available genetic, segregation, structural, and computational evidence supports the potential functional relevance of p.Cys151Ser but remains insufficient to classify it as likely pathogenic in the absence of direct protein-level functional evidence or additional independent genetic evidence. |

**Table S6. Results of the 12-item odor identification test.** Correct indicates that the target odor was identified correctly. For incorrect responses, the participant's selected alternative odor is reported after a colon.

**A. Summary of individual scores**

| **Individual** | **Clinical status** | **Left nostril (correct/12)** | **Right nostril (correct/12)** | **Bilateral (correct/12)** |
| --- | --- | --- | --- | --- |
| Individual III-3 | Affected | 4 | 6 | 4 |
| Individual III-2 | Affected | 4 | 4 | 4 |
| Individual II-2 (mother) | Unaffected carrier | 11 | 11 | 10 |

Scores represent the number of correctly identified odors out of 12.

**B. Detailed responses: Individual III-3**

| **No.** | **Target odor** | **Left nostril** | **Right nostril** | **Bilateral testing** |
| --- | --- | --- | --- | --- |
| 1 | Orange | Correct | Incorrect: pineapple | Correct |
| 2 | Leather | Incorrect: smoke | Incorrect: smoke | Incorrect: grass |
| 3 | Cinnamon | Incorrect: vanilla | Correct | Correct |
| 4 | Mint | Incorrect: onion | Correct | Incorrect: chives |
| 5 | Banana | Incorrect: cherry | Correct | Incorrect: cherry |
| 6 | Lemon | Correct | Correct | Incorrect: melon |
| 7 | Licorice | Incorrect: gummy candy | Correct | Correct |
| 8 | Coffee | Correct | Incorrect: cigarette | Incorrect: cigarette |
| 9 | Clove | Incorrect: cinnamon | Incorrect: mustard | Incorrect: mustard |
| 10 | Pineapple | Incorrect: plum | Incorrect: peach | Incorrect: peach |
| 11 | Rose | Incorrect: raspberry | Incorrect: chamomile | Incorrect: chamomile |
| 12 | Fish | Correct | Correct | Correct |

Total score: left nostril 4/12; right nostril 6/12; bilateral testing 4/12.

**C. Detailed responses: Individual III-2**

| **No.** | **Target odor** | **Left nostril** | **Right nostril** | **Bilateral testing** |
| --- | --- | --- | --- | --- |
| 1 | Orange | Correct | Correct | Correct |
| 2 | Leather | Incorrect: grass | Incorrect: grass | Incorrect: grass |
| 3 | Cinnamon | Incorrect: vanilla | Correct | Correct |
| 4 | Mint | Incorrect: spruce | Incorrect: spruce | Incorrect: spruce |
| 5 | Banana | Incorrect: coconut | Incorrect: coconut | Incorrect: coconut |
| 6 | Lemon | Incorrect: cherry | Incorrect: cherry | Incorrect: cherry |
| 7 | Licorice | Correct | Incorrect: gummy candy | Incorrect: gummy candy |
| 8 | Coffee | Incorrect: smoke | Incorrect: smoke | Incorrect: smoke |
| 9 | Clove | Correct | Correct | Correct |
| 10 | Pineapple | Incorrect: peach | Incorrect: plum | Incorrect: plum |
| 11 | Rose | Incorrect: cherry | Incorrect: chamomile | Incorrect: chamomile |
| 12 | Fish | Correct | Correct | Correct |

Total score: left nostril 4/12; right nostril 4/12; bilateral testing 4/12.

**D. Detailed responses: Individual II-2 (mother)**

| **No.** | **Target odor** | **Left nostril** | **Right nostril** | **Bilateral testing** |
| --- | --- | --- | --- | --- |
| 1 | Orange | Correct | Correct | Correct |
| 2 | Leather | Correct | Correct | Correct |
| 3 | Cinnamon | Incorrect: vanilla | Incorrect: vanilla | Incorrect: vanilla |
| 4 | Mint | Correct | Correct | Correct |
| 5 | Banana | Correct | Correct | Incorrect: cherry |
| 6 | Lemon | Correct | Correct | Correct |
| 7 | Licorice | Correct | Correct | Correct |
| 8 | Coffee | Correct | Correct | Correct |
| 9 | Clove | Correct | Correct | Correct |
| 10 | Pineapple | Correct | Correct | Correct |
| 11 | Rose | Correct | Correct | Correct |
| 12 | Fish | Correct | Correct | Correct |

Total score: left nostril 11/12; right nostril 11/12; bilateral testing 10/12.

**Table S7. Differential expression of selected candidate genes.** Gene-level RNA-seq results for affected individuals (study group; q1) compared with the control (control group; q2). The table includes normalized expression values, log₂ fold changes, raw p-values, and adjusted p-values obtained for the comparison K1_KN_and_K3_KA_vs_WP_K2_ctrl. Genes with an adjusted p-value < 0.05 were considered statistically significant. RNA-seq data are available in the GEO under accession number GSE318901.

q1=K1_KN and K3_KA

q2=WP2_K2_ctrl

| **Test_Id** | **Gene** | ***locus*** | **Study group** | **Controlgroup** | **status** | **value_1** | **value_2** | **log2(fold_change)** | **test_stat** | **p_value** | **q_value** | **significant** |
| --- | --- | --- | --- | --- | --- | --- | --- | --- | --- | --- | --- | --- |
| *ENSG00000011201.13* | *ANOS1* | *chrX:8528873-8732195* | q1 | q2 | NOTEST | 0.0585249 | 0.0167227 | -1.80725 | 0 | 1 | 1 | no |
| *ENSG00000163492.17* | *CCDC141* | *chr2:178521182-179051697* | q1 | q2 | OK | 9.97506 | 11.046 | 0.14713 | 0.103724 | 0.8708 | 0.999655 | no |
| *ENSG00000139318.9* | *DUSP6* | *chr12:89347234-90143524* | q1 | q2 | OK | 141.694 | 105.598 | -0.424194 | -0.903051 | 0.2885 | 0.999655 | no |
| *ENSG00000101596.18* | *SMCHD1* | *chr18:2652167-2833065* | q1 | q2 | OK | 825.288 | 584.11 | -0.498656 | -0.941664 | 0.1933 | 0.999655 | no |
| *ENSG00000141002.21* | *TCF25* | *chr16:89873565-89938761* | q1 | q2 | OK | 90.7099 | 83.4293 | -0.120706 | -0.330037 | 0.6922 | 0.999655 | no |

**Table S8. qRT-PCR validation of *PROK2* and *DUSP6* expression in affected individuals and controls.**

| **Target gene** | **Sample** | **Clinical status** | **Reference gene** | **Mean Ct target**  **± SD** | **Mean Ct reference ± SD** | **ΔCt** | **ΔΔCt** | **Relative expression**  **2−ΔΔCt** |
| --- | --- | --- | --- | --- | --- | --- | --- | --- |
| *PROK2* | K3_KA | Affected | *HPRT1* | 17.96 ± 0.02 | 22.69 ± 0.06 | −4.74 | −0.72 | 1.65 |
| *PROK2* | K1_KN | Affected | *HPRT1* | 17.03 ± 0.01 | 22.62 ± 0.07 | −5.59 | −1.58 | 2.98 |
| *PROK2* | Control | Unaffected | *HPRT1* | 18.79 ± 0.04 | 22.81 ± 0.06 | −4.02 | 0.00 | 1.00 |
| *DUSP6* | K3_KA | Affected | *HPRT1* | 18.61 ± 0.05 | 22.87 ± 0.05 | −4.26 | −0.21 | 1.15 |
| *DUSP6* | K1_KN | Affected | *HPRT1* | 18.79 ± 0.01 | 22.65 ± 0.06 | −3.86 | 0.20 | 0.87 |
| *DUSP6* | Control | Unaffected | *HPRT1* | 19.15 ± 0.01 | 23.20 ± 0.11 | −4.06 | 0.00 | 1.00 |
| *DUSP6* | K3_KA | Affected | *TBP* | 18.61 ± 0.05 | 22.71 ± 0.21 | −4.10 | 0.01 | 1.00 |
| *DUSP6* | K1_KN | Affected | *TBP* | 18.79 ± 0.01 | 22.75 ± 0.15 | −3.96 | 0.15 | 0.90 |
| *DUSP6* | Control | Unaffected | *TBP* | 19.15 ± 0.01 | 23.25 ± 0.14 | −4.11 | 0.00 | 1.00 |

Ct values are presented as the mean ± SD of two technical replicates. ΔCt was calculated as Ct_target − Ct_reference. The unaffected control sample was used as the calibrator, and ΔΔCt was calculated as ΔCt_sample − ΔCt_control. Relative expression was calculated using the 2−ΔΔCt method. PROK2 expression was normalized to HPRT1. DUSP6 expression was independently evaluated using HPRT1 and TBP as reference genes.

**Figure S1. Exon-level analysis of *PROK2* expression**. Differential exon usage in *PROK2* was assessed between the study group and the control group using DEXSeq. (A) Exon-level expression plot generated with the plotDEXSeq function. The red and blue lines represent exon-level expression profiles in the control and study groups, respectively. No statistically significant differences in exon usage were detected. (B) Summary of the exon-level statistical analysis. These results indicate that increased *PROK2* expression was not accompanied by detectable alterations in exon usage.
